# Regionalized dynamic climate series for ecological climate impact research in modern controlled environment facilities

**DOI:** 10.1002/ece3.8371

**Published:** 2021-11-20

**Authors:** Bálint Jákli, Roman Meier, Ulrike Gelhardt, Margaret Bliss, Ludger Grünhage, Manuela Baumgarten

**Affiliations:** ^1^ Land Surface‐Atmosphere Interactions Technical University of Munich Freising Germany; ^2^ Interdepartmental research facility TUMmesa Technical University of Munich Freising Germany; ^3^ MeteoSolutions GmbH Darmstadt Germany; ^4^ Department of Plant Ecology Justus‐Liebig‐Universität Giessen Gießen Germany

**Keywords:** climate change, climate simulations, controlled environment, ecotron, RCP scenarios, TUM*mesa*

## Abstract

Modern controlled environment facilities (CEFs) enable the simulation of dynamic microclimates in controlled ecological experiments through their technical ability to precisely control multiple environmental parameters. However, few CEF studies exploit the technical possibilities of their facilities, as climate change treatments are frequently applied by static manipulation of an inadequate number of climate change drivers, ignoring intra‐annual variability and covariation of multiple meteorological variables. We present a method for generating regionalized climate series in high temporal resolution that was developed to force the TUM*mesa* Model EcoSystem Analyzer with dynamic climate simulations. The climate series represent annual cycles for a reference period (1987–2016) and the climate change scenarios RCP2.6 and RCP8.5 (2071–2100) regionalized for a climate station situated in a forested region of the German Spessart mountains. Based on the EURO‐CORDEX and ReKliEs‐DE model ensembles, typical annual courses of daily resolved climatologies for the reference period and the RCP scenarios were calculated from multimodel means of temperature (t_a_), relative humidity (rh), global radiation (R_g_), air pressure (P), and ground‐level ozone and complemented by CO_2_. To account for intra‐annual variation and the covariability of multiple climate variables, daily values were substituted by hourly resolved data resampled from the historical record. The resulting present climate Test Reference Year (TRY) well represented a possible annual cycle within the reference period, and expected shifts in future mean values (e.g., higher t_a_) were reproduced within the RCP TRYs. The TRYs were executed in eight climate chambers of the TUM*mesa* facility and—accounting for the technical boundaries of the facility—reproduced with high precision. Especially, as an alternative to CEF simulations that reproduce mere day/night cycles and static manipulations of climate change drivers, the method presented here proved well suited for simulating regionalized and highly dynamic annual cycles for ecological CEF studies.

## Graphical abstract

Modern controlled environment facilities enable dynamic simulation of present and future climate scenarios for ecological climate impact research. We present a method for the generation of regionalized high‐resolution climate series for present and RCP‐based climate scenarios. 6‐months‐segments of each scenario were tested for applicability and reproducibility in the TUM*mesa* Model EcoSystem Analyser.

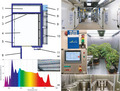

## 1 INTRODUCTION

Climate change alters ecosystem functioning worldwide with severe consequences for biodiversity, ecosystem services and for the well‐being of humankind (IPBES, 2019; IPCC, 2013). In order to understand the impact of climate change on terrestrial ecosystems and to develop sustainable management strategies, scientific experiments with controlled modification of environmental drivers are essential. Previous studies in this field have largely been limited to the experimental manipulation of one or a limited set of meteorological drivers. Thus far, particular focus has been on the modification of atmospheric CO_2_, air temperature (t_a_), as well as precipitation and tropospheric ozone (O_3_) concentration (reviewed by Ainsworth & Long, 2005; Ainsworth et al., 2012; Beier et al., 2012; Lin et al., 2010). Such experiments are helpful in identifying general mechanisms of physiological and ecological responses (De Boeck et al., 2015), but ignore the covariation of multiple, physically interdependent variables. Responses by ecological systems to the simultaneous manipulation of multiple environmental drivers are unique and cannot be directly extrapolated from the response to each of the drivers manipulated individually (Ogle et al., 2021; Suzuki et al., 2014). For example, monthly mean surface temperatures and precipitation are tightly linked (Trenberth & Shea, 2005), and periods of reduced soil moisture availability covary with temperature and high light intensity (Suzuki et al., 2014).

Even large‐scale field studies only allow the rough manipulation of a limited number of environmental variables (e.gBurkart et al., 2009; Eastburn et al., 2010) and should be ideally embedded in a framework including experiments in controlled environment facilities (CEFs) and computational modeling (Hanson & Walker, 2020; Roy et al., 2020a). In this context, modern CEFs offer the possibility to precisely and dynamically regulate many environmental conditions. In addition to the common control of t_a_, relative humidity (rh), and CO_2_, modern LED lighting provides a multispectral, dynamic light regulation. Some facilities accurately dose and monitor O_3_, NO_X_, or stable isotopes (^13^C, ^18^O). Nonmeteorological parameters such as soil moisture and nutrient supply are also commonly manipulated in lysimeter planters and automatically controlled. Given the high quantity of meteorological and ecological parameters that can be independently controlled, these CEFs are commonly referred to as “ecotrons” (Roy et al., 2020a).

Nevertheless, only a limited number of CEF studies exploit the technical possibilities of their facilities. Leisner et al. (2018) showed that in 80% of 57 reviewed agricultural CEF studies, static day and night temperatures were applied. For climate change scenarios, both temperature and CO_2_ were typically increased in discrete steps, more or less based on previous experience with thresholds and tipping points, rather than according to model predictions (Leisner et al., 2018). This simplified representation of future climatic conditions may substantially limit the validity of controlled growth experiments with respect to the regional impacts of climate change on ecosystems.

In order to overcome this shortcoming, attempts have been made to incorporate seasonal and diurnal variability in present and future climate (FC) scenarios employed in CEF experiments. Thompson et al. (2013) summarized those attempts as *increment studies*, *extreme event studies*, and *down‐scaled climate studies*. According to the authors, increment studies aim to preserve diurnal meteorological dynamics by imposing a fixed incremental increase on a natural meteorological quantity. This approach assumes a uniform shift over the entire diurnal and seasonal cycle (e.gGhirardo et al., 2020; Hayes et al., 2019), for example, a model predicted mean increase in t_a_. However, the general consensus maintains that FC will not only be characterized by a shift in mean meteorological quantities, but will also see a significant increase in the variability, intensity, frequency, and duration of extreme meteorological events such as droughts or heavy rainfall (Jentsch et al., 2007). There is evidence that extreme weather events coupled with gradual climate trends may push ecosystems beyond their tipping‐points (Harris et al., 2018). For CEF studies, this implies extreme intensities of a meteorological driver to be temporally superimposed on an existing climate series (Roy et al., 2016; Thompson et al., 2013). Both increment and extreme event studies preserve the natural temporal variability of a meteorological quantity, without considering interdependency and co‐variability of multiple environmental quantities.

In recent years, it has been attempted to reproduce climatic conditions of present climate (PC) and FC scenarios in CEF experiments as realistically as possible, taking into account the co‐variability of multivariate drivers. This requires global climate model (GCM) output to be adapted for application in controlled environments. GCMs are usually available at relatively coarse temporal resolution. However, in order to capture not only large‐scale seasonal, but also regional diurnal dynamics, CEF studies require climate series with high temporal (hours) and spatial (a few kilometers) resolution. This can be achieved by statistical or dynamic downscaling of GCMs to the regional scale (Giorgi & Gutowski, 2015; Wilby et al., 1998).

Thompson et al. (2013) developed one of the first ecological applications of statistical downscaling for CEF experiments. To generate valid temperature series for the year 2100, the authors combined the MIROC GCM with statistical information from real observations, and then employed a stochastic weather generator to obtain data at an hourly resolution. Similarly, Roy et al. (2016) used global information from the ARPEGEv4 GCM to simulate realistic 2040–2060 climate forcing at the Montpellier CNRS Ecotron facility. To regionalize the GCM output, the authors used the multivariate statistical downscaling method developed by Boé et al. (2006). The authors generated regionalized climate series through conditional resampling using data from the historical record to match statistical properties of a GCM output. By resampling, the natural variability and the covariance within multivariate climate series was preserved. Recently, Vanderkelen et al. (2020) presented an elegant method for forcing the UHasselt Ecotron units directly with the output of a single well‐defined combination of a GCM with a regional climate model (RCM). Their method required the sophisticated identification of the best‐performing GCM:RCM simulation for the mid‐21st century from the Coordinated Downscaling Experiment‐European Domain (EURO‐CORDEX) model ensemble for the site of the ecotron. Their method not only accounts for the co‐variation between climatic variables and their projection in variability, but also represents extreme weather events. In contrast, Leisner et al. (2018) used an ensemble average across six different GCM:RCM combinations from the NARCCAP downscaling program to simulate a mean temperature scenario of the Eau Clair region, USA, for the years 2040–2060 in their CEF units.

Averaging across model ensembles may smooth out extreme events present in individual GCM:RCM runs. However, individual outputs within a model ensemble may differ considerably, representing a fundamental resource for studying the range of possible climate responses—including extreme events—to a given forcing (Field et al., 2012). In contrast, multimodel averages allow a robust simulation of the mean meteorological conditions of a specific climate scenario. Therefore, they are particularly useful for comparing the mean responses of ecological systems among various FC scenarios. In this study, we present a methodological approach that allows the generation of robust, high‐resolution dynamic climate series for application in ecological CEF studies based on GCM:RCM ensemble means. For this purpose, we selected GCM:RCM combinations from the EURO‐CORDEX (Jacob et al., 2014) and ReKliEs‐DE (Regional Climate change Ensemble simulations for Germany, Hübener et al., 2017) ensembles. For each of the nine selected GCM:RCM combinations, simulations of a reference period (1987–2016) and a future period (2071–2100) were selected, the latter forced by the opposing representative concentration pathway scenarios RCP2.6 (van Vuuren et al., 2011) and RCP8.5 (Riahi et al., 2011). Ensemble means were formed for each scenario and averaged over nine 0.11°‐grid‐points covering the study area. Subsequently, homogenous weather segments were identified in each time series and replaced with historical records of t_a_, rh, global radiation (R_g_), and O_3_ in one‐hour‐resolution using a resampling method. The generated annual Test Reference Years (TRYs) were complemented by CO_2_ series and used to force the eight climate chambers of the Model Ecosystem Analyzer TUM*mesa* at the Technical University of Munich for six consecutive months each year from 2019 to 2021 within the “valORTree” project. First, we describe the methodological approach for generating the TRYs and then evaluate the applicability of the TRYs for ecological climate impact research in the TUM*mesa* CEFs.

## 2 MATERIAL AND METHODS

### 2.1 TUM*mesa*

**FIGURE 1:**
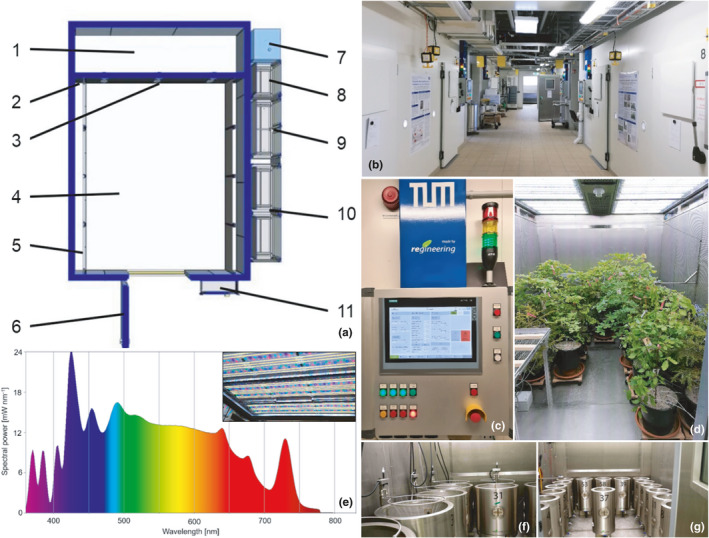
Top view of a TUM*mesa* experimental chamber (a). 1: utility room, 2: air passage, 3: maintenance access, 4: experimental space, 5: perforated plate for air inlet, 6: door, 7: evaporator, 8: LED controls, 9: glycol controls, 10: power controls, 11: control panel. TUM*mesa* experimental facility (b). Control panel (c). Beech and spruce trees inside an experimental chamber (d). LED spectrum (without UV) and panels (e). Chamber equipped with nine 200‐L‐lysimeters (f). Chamber equipped with sixteen 50‐L‐lysimeters (g). (a), (e), (f), (g) courtesy of ***re*** gineering GmbH

TUM*mesa* is an interdepartmental research institution of the Technical University of Munich and one of the most modern publicly operated CEFs in Germany. The facility features eight identical experimental walk‐in chambers (Figure 1) engineered by ***re*** gineering GmbH (Pollenfeld, Germany) that allow the generation of a range of ecological conditions (e.g., Yang et al., 2019; Zytynska et al., 2020), with precise control of t_a_, rh, light, CO_2_, and O_3_, as well as manipulation of soil moisture, soil temperature and nutrient supply in various planters and lysimeters. Each climate chamber provides an experimental space of 2.4 × 3.2 × 2.2 m (W × D × H) and is equipped with the following features: air inlet/outlet/circulation unit, cooling and heating register, steam generator and humidifier unit, CO_2_ and O_3_ fumigation, LED lighting, ^13^CO_2_ labeling system as well as automatic irrigation/fertigation coupled with an automatic weighing system for planters and lysimeters. Preconditioned air is uniformly directed into the experimental space across the fully perforated sidewalls, producing an average wind speed of <0.1 m s^−1^. All aggregates, supply lines, and control cabinets are installed in immediate vicinity to the chambers. The LED system (Vossloh Schwabe, Urbach, Germany) comprises 10 individually controllable sub‐systems that allow near‐realistic sunlight and PAR simulation within a spectral range spanning UV‐B to far‐red. The LED system provides a maximum PPFD of >1500 µmol m^−2^ s^−1^ in one meter distance from the panels. By default, LEDs are operated with 55% of maximum capacity, providing a PPFD of >800 µmol m^−2^ s^−1^. An overview of the technical specifications and limitations of TUM*mesa* is presented in Table A1 (see Appendix S1) and by Roy et al. (2020).

### 2.2 “valORTree” project and study area

**FIGURE 2:**
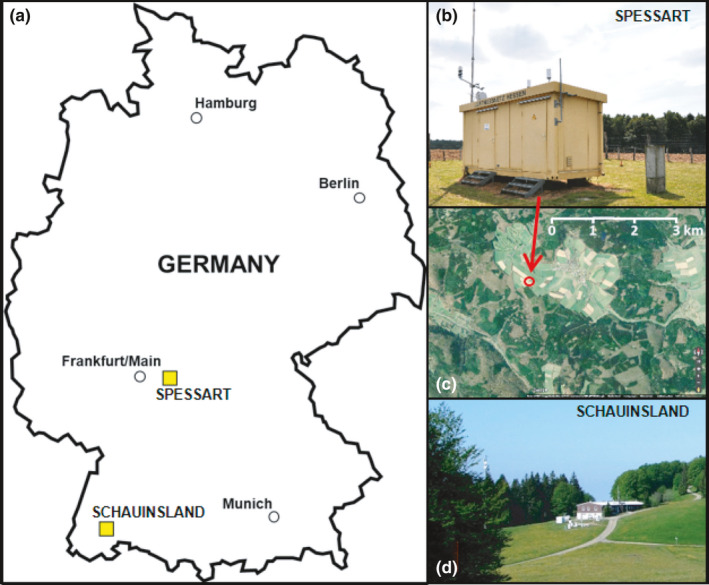
Locations of the meteorological stations “Spessart” and “Schauinsland” in Germany (a). Views of the “Spessart“ station (b) situated in a forested area of the Spessart mountains (c) and the “Schauinsland” station (d). Courtesy of the Hessian National Office for Environment and Geology (HLNUG) (b) and of C. Zinsius, German Environment Agency (UBA) (d)

This study is part of the "valORTree" project. In this project, O_3_ dose‐response functions for two economically important tree species European beech (*Fagus sylvatica* L.) and Norway spruce (*Picea abies* (L.) H. Karst.) were established under controlled conditions using a gradient approach. Furthermore, the future O_3_ risk potential was evaluated for the RCP2.6 and RCP8.5 climate change scenarios and the parametrization of the FO3REST ozone deposition model was updated (see Bender et al. (2015) for model description). Briefly, 10 beech and 10 spruce trees were arranged in each of the eight TUM*mesa* climate chambers, (Figure 1d). Trees, including a 20‐L soil monolith, were harvested from a naturally regenerating forest. Tree age ranged 5–10 years with an average tree height of 110 ± 18 cm for beech and 76 ± 11 cm for spruce. Generating robust dose‐response functions required continuous measurements of stomatal O_3_ uptake. In order to allow realistic diurnal dynamics of stomatal regulation throughout the experiment, external meteorological variables were simulated as dynamically as possible while considering interdependencies between multiple variables. Therefore, it was crucial to generate robust, self‐consistent multivariate climate series that reproduced the mean seasonal and diurnal dynamics of PC and FC scenarios at a representative study site. The study site was selected based on three criteria: (i) availability of long‐term meteorological records in hourly resolution, including accurate measurements of tropospheric O_3_ concentration; (ii) location of the corresponding climate station in a forested area characterized by beech and spruce stands; (iii) free exposure and no immediate influence of anthropogenic combustion processes. Based on these criteria, the atmospheric measurement station “Spessart” (Jossgrund‐Lettgenbrunn, Germany, Code DEHE026, 497 m asl, 50°09′52.0″N 9°23′58.0″E) was selected (Figure 2). The station is located in the German Spessart mountain range and is operated by the Hessian National Office for Environment and Geology since 1986. Meteorological data, along with air pollutants, are recorded at the station at 3.5 m above ground level. For further processing, t_a_, rh, P, R_g_, and O_3_ records of the 1987–2016 reference period were selected. Hereafter, the term *reference climate* (RC) refers to the 30‐years average, daily resolved annual course of the parameters measured for the reference period at the climate station.

As CO_2_ concentrations were not measured at the “Spessart” station, data from the nearest CO_2_ monitoring station “Schauinsland” (operated by the German Federal Environment Agency (UBA), 1205 m.a.s.l., 47°54′49.7″N 7°54′27.9″E) were used. Quality control and gap filling were performed to obtain continuous hourly time series from 1987 to 2016. Precipitation was not considered because the objectives of the valORTree project required adequate soil moisture availability. However, to account for possible reduced precipitation levels and the resulting limited soil moisture availability, FC TRYs were replicated with a period of reduced soil moisture.

### 2.3 Data analysis

Data processing, analysis, and visualization were performed with R v3.5.3 (R Core Team, 2020). Frequently used packages were *corrplot* v 0.77 (Wei & Simko, 2017), *dplyr* v.0.8.3 (Wickham et al., 2019), *plotrix* v3.7‐6 (Lemon et al., 2015), and *plyr* v1.8.4 (Wickham, 2011). Standard deviation (*SD*) is given as an indicator of deviation from mean values. Climate extreme indices (Zhang et al., 2005, 2011) were calculated using the R package *climdex.pcic* v1.1‐11 (Bronaugh, 2020).

### 2.4 Generation of regionalized TRY for PC, RCP2.6 and RCP8.5

**FIGURE 3:**
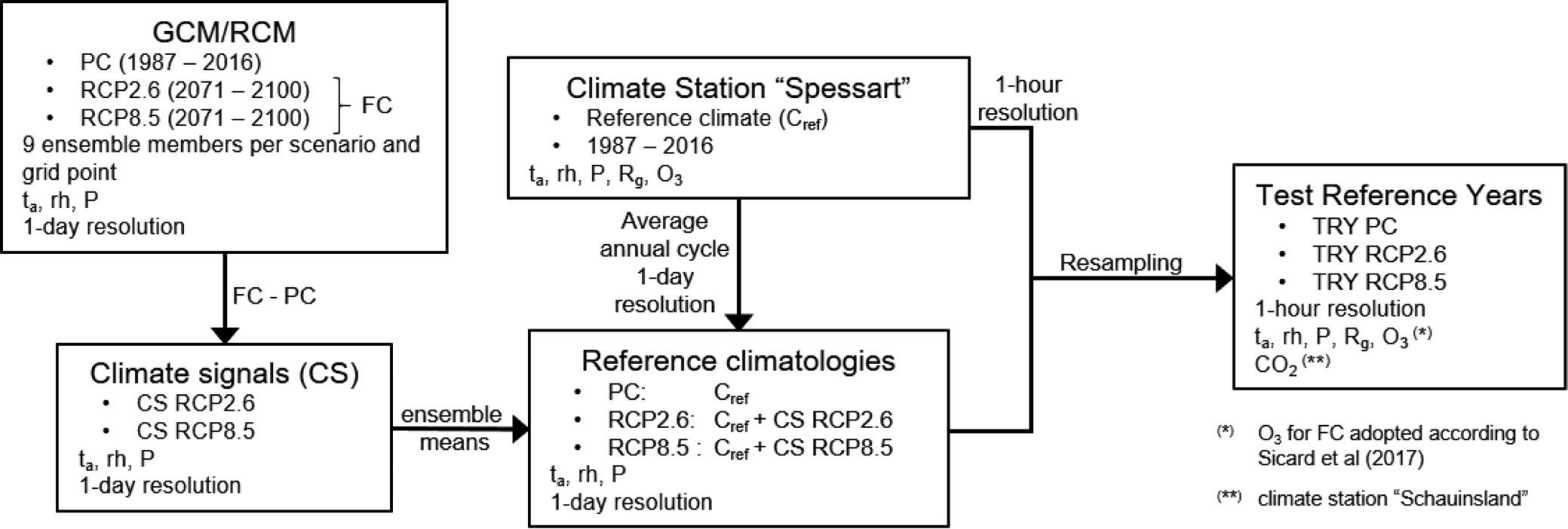
Schematic representation of the workflow for generating the TRY. Ensembles of regional climate simulations were obtained for PC and the FC scenarios RCP2.6 and RCP8.5 from GCM:RCM combinations. Next, CSs were calculated by subtracting PC variables from FC. The RC for PC was calculated as the average annual cycle of climate records at the station “Spessart.” Reference climatologies for the RCPs were computed by adding the respective ensemble mean climate signal to the RC. Finally, characteristic weather segments recorded at “Spessart” were resampled from historical record to match statistical properties of the reference climatologies

The methodology to generating TRYs that represent annual courses of PC (1987–2016) and FC (2071–2100) is based on DWD (2017) and was further developed and implemented by MeteoSolutions GmbH (Darmstadt, Germany) (Gelhardt et al., 2021). The procedure includes the computation of climate signals (CSs) for the FC scenarios RCP2.6 and RCP8.5, the generation of reference climatologies and resampling from the historical record (Figure 3).

#### 2.4.1 Data processing

The EURO‐CORDEX initiative provides ensembles of regional climate simulations for the European domain (Jacob et al., 2014). The simulations combine a GCM from the Coupled Model Intercomparison Project Phase 5 (CMIP5) with a RCM or an empirical statistical downscaling (ESD) method. Wheras, dynamically downscaled RCMs are forced by GCMs at their initial and lateral boundaries to produce climate simulation data on a fine regional scale (Giorgi & Gutowski, 2015), ESDs are based on transfer functions that connect observations on the GCM scale to regional records (Kreienkamp et al., 2019). The EURO‐CORDEX simulations use the EUR‐11 domain at 0.11° grid spacing and cover the historical periods 1989–2008 and 1951–2005, as well as the future scenarios RCP2.6, 4.5, and 8.5 for 2006–2100 (Jacob et al., 2020). The German ReKliEs‐De project complements EURO‐CORDEX with an ensemble of 12 dynamically downscaled GCM:RCM simulations for the European domain and 16 statistically downscaled simulations exclusively for Germany (Hübener et al., 2017, 2018). ReKliEs‐De simulations use the same 0.11° grid as EURO‐CORDEX for the historic period 1971–2000 and the RCP scenarios 2.6 and 8.5 for 2071–2100. For this study, GCM:RCM/ESD combinations were selected from the ReKliEs‐DE ensemble where simulations for both RCP2.6 and RCP8.5 were availiable (Figure A2 in Appendix S1). Furthermore, some combinations were technically excluded (see Appendix S1). The following nine GCM:RCM combinations were used for further analysis (WETTREG is the only ESD, all other downscaling methods were of dynamic type): EC‐EARTH: CCLM, RCA4, RACMO; HadGEM2: RCA4, RACMO; MPI‐ESM: CCLM, REMO, WETTREG, RCA4.

Simulation data of near‐surface t_a_, near‐surface rh (if not available: near‐surface specific humidity) and surface P were downloaded in daily resolution from the Earth System Grid Federation data portal. Data of a 3 × 3‐grid‐points subarea (lat 50.0°–50.3°, lon 9.2°–9.6°) with the climate station as the center point were extracted from the downloaded data set. As an exception, the coordinate system of REMO with MPI‐ESM forcing is shifted by half a grid box, such that a 2 × 2‐grid‐point subarea was used (lat 49.9°–50.2°, lon 9.3°–9.6°). Near‐surface specific humidity (*q*)—and not rh—was available for the model combinations EC‐EARTH:CCLM, MPI‐ESM:CCLM, MPI‐ESM:REMO, and HadGEM2:RCA4. *q* was converted to rh by relating atmospheric vapor pressure (*e*) to saturation vapor pressure (*E*)

(1) $$rh=\frac{e}{E}\cdot 100$$

*E* and *e* were calculated according to Bolton (1980):

(2) $$e=\frac{q\cdot P}{0.378\cdot q+0.622}$$

(3) $$E=6.122e^{\frac{17.62\cdot {\text{t}}_{\text{a}}}{243.12+{\text{t}}_{\text{a}}}}$$

Air pressure from the MPI‐ESM:CCLM model combination was available in 3‐hr resolution and was converted to daily mean values. MPI‐ESM:WETTREG was the only combination involving an ESD. WETTREG links global circulation patterns derived from a GCM with a stochastic weather‐generating algorithm that produces random weather series (Kreienkamp et al., 2013). To improve the statistical validity of the data produced by the weather generator, a total of 10 model runs of the MPI‐ESM:WETTREG combination were averaged.

#### 2.4.2 Computaion of CSs

**FIGURE 4:**
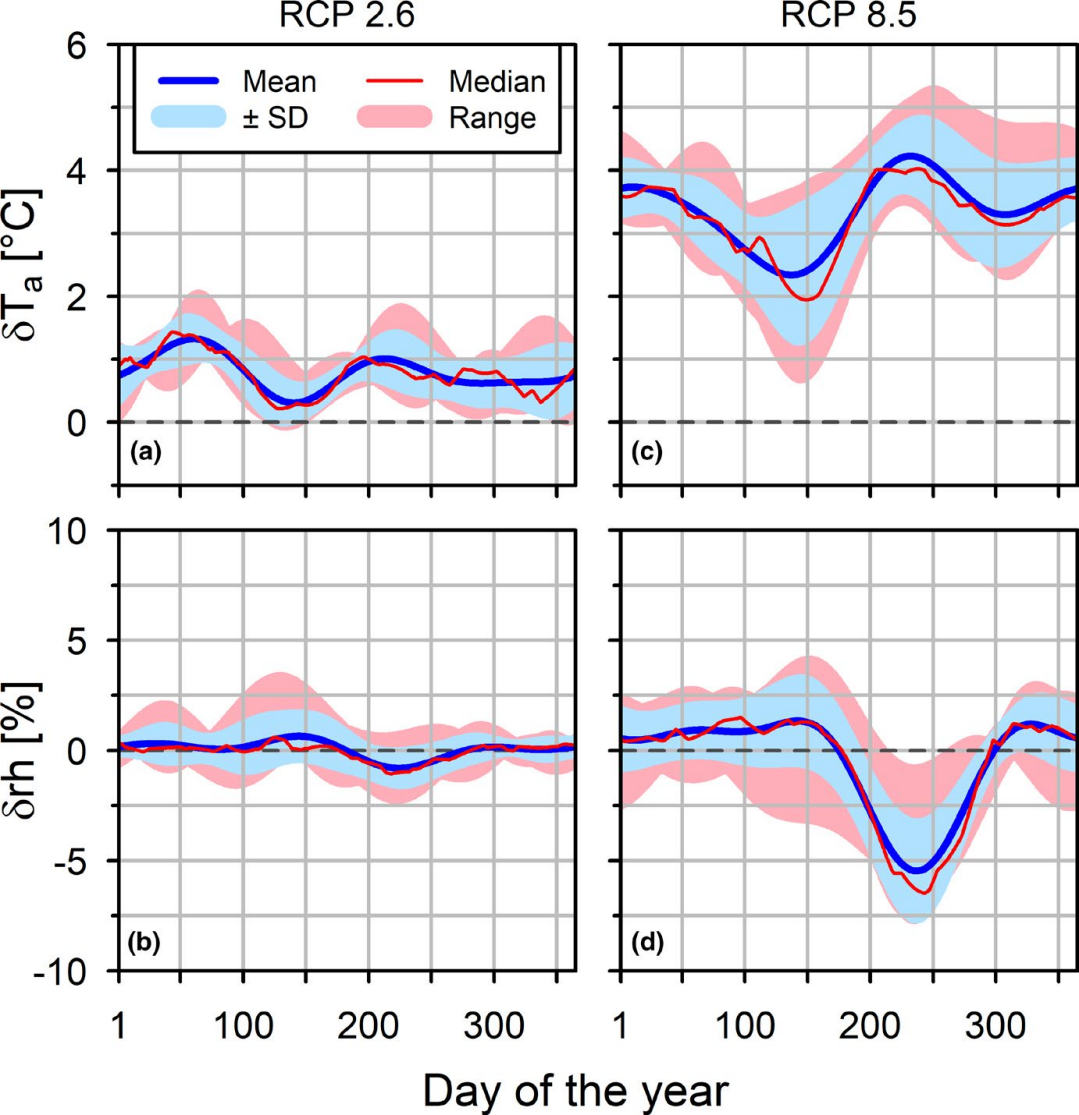
Annual course of climate signals calculated for the RCP2.6 (a,b) and RCP8.5 (b,c) scenarios at the climate station “Spessart.” The climate signals indicate the difference between present and predicted daily averages of air temperature (δT_a_) (a,c) and relative humidity (δrh) (b,d). Mean values ± SD, median and range of nine simulated climate series per scenario and grid point are shown

Future climate simulations were independently generated for both RCP scenarios. PC was simulated by combining data from historic climate projections (1987–2005) and FC projections (2005–2016). For the latter, data were simulated for both RCP2.6 and RCP8.5 and averaged. Data from all simulations were fitted to a standard calendar of 365 days while average annual courses of selected climate variables were computed in daily resolution. Noise‐reduction was performed on each time series using Fast Fourier Transformation, through which frequencies higher than third order were eliminated. CSs for t_a_ and rh were obtained by calculating the difference between t_a_ and rh of the PC and the FC simulations for each ensemble member and grid point. The CSs of both RCP scenarios were averaged over all ensemble members and grid points. Thus, multimodel mean annual climatologies of the CSs with data in daily resolution were obtained for RCP2.6 and RCP8.5 (Figure 4), which were added to the RC recorded at the “Spessart” station. RC of t_a_, rh, and R_g_ was defined as the reference climatology (RC) for PC.

#### 2.4.3 Resampling

A resampling method (DWD, 2017; Gelhardt et al., 2021) was applied in order to compute characteristic TRYs of t_a_, rh, P, R_g_, and O_3_ in hourly resolution for PC, RCP2.6, and RCP8.5. Homogeneous weather segments recorded at “Spessart” from 1987 to 2016 in daily resolution were resampled in order to match statistical properties of the corresponding period of the reference climatologies. For the first segment starting from 1 January, all possible 10–30 days long segments of the 30‐year record (=630 segments) were tested against the corresponding segments of the reference climatologies with respect to the differences in mean values of t_a_, rh, R_g_, and in the *SD* of t_a_, as well as (for all segments but the first) the absolute t_a_ difference to the preceding segment. The differences were listed in ascending order and scores staring from 0 (smallest difference) were assigned. Scores for the difference in mean t_a_ were multiplied with the factor 0.3 and for the *SD* of t_a_ with 0.7. Subsequently, the scores for each possible segment were summed and the weather segment achieving the lowest score was chosen. This procedure was repeated for the following segments until 31 December. Finally, the daily data of the recombined weather segments were replaced by the corresponding hourly records of t_a_, rh, P, R_g_, and O_3_. In order to smooth the transition between individual segments, t_a_, rh, P, and O_3_ (not R_g_) were linearly interpolated between 8 h to the end of one segment and 8 h after the beginning of the next segment.

The FC O_3_ series generated for the RCPs by this method reflect the impact of meteorological quantities on O_3_ levels under present atmospheric conditions, but do not respect possible changes in future tropospheric trace gas composition. However, O_3_ formation and depletion are highly controlled by the presence of ground‐level NO_X_, CH_4_, and VOCs, the concentrations of which will evolve differently under the RCP scenarios. According to Sicard et al. (2017), northern hemisphere O_3_ concentrations are expected to decline by 25% until 2100 for RCP2.6 and to increase by 11.5% for RCP 8.5. O_3_ series obtained by resampling were adjusted accordingly: the series were normalized to the respective annual mean O_3_ to obtain relative values, which were then multiplied by the long‐term average of the reference station reduced by 25% (RCP2.6) or increased by 11.5% (RCP8.5).

The TRYs were complemented by time series of tropospheric CO_2_ concentration adapted for the different climate scenarios. First, a relative annual CO_2_ series was calculated by normalizing the average annual CO_2_ course recorded from the years 1987–2016 at the climate station “Schauinsland” on the respective average annual concentration of CO_2_. Representative time series for PC, RCP2.6, and RCP8.5 were generated by multiplying the relative hourly CO_2_ series by 375 ppm (30‐years average from “Spessart” station), 421 ppm and 936 ppm (simulated for RCP2.6 and RCP8.5 in 2100, see Meinshausen et al., 2011).

### 2.5 Simulation of PC, RCP2.6 and RCP8.5 in TUM*mesa*

The capacity of the TUM*mesa* CEF to operate complex climate simulations by controlling parameters in accordance with the prescribed values during long‐term operation was investigated during the first experimental campaign of the three‐year project “valORTree.” Data were recorded on 161 operational days between 16 April and 29 September 2019. Chambers C1 and C2 executed TRY RCP8.5, C3 and C4 executed TRY RCP2.6 and C5, C6, C7, and C8 executed TRY PC with an additional O_3_ gradient. Throughout the experiment, soil moisture was monitored by custom made TDR sensors and was maintained at ~80% of field capacity by adjusting the drip irrigation time. Soil moisture of C2 and C4 was reduced to 30% of field capacity for ten days in August.

Prior to the implementation of the TRYs into the TUM*mesa* control program, the climate series were adjusted (Table 1) to meet the climate chambers’ technical requirements (Table A1 in Appendix S1) or for experimental purpose. Air temperatures exceeding 30°C were limited to 30°C. Day/night t_a_ below 10/4°C was increased to 10/4°C and rh was limited to 75%/90%. R_g_ was converted to PPFD by multiplying R_g_ by a month‐specific empirical conversion factor ranging from 1.90 to 2.10 (Grünhage & Haenel, 2008). PPFD between 600 µmol m^−2^ s^−1^ and the maximum intensity of 2030 µmol m^−2^ s^−1^ were scaled to the range from 600 to 800 µmol m^−2^ s^−1^. PPFDs < 24 µmol m^−2^ s^−1^ were increased to 24 µmol m^−2^ s^−1^ due to minimum requirements of the LEDs (Figure A1 in Appendix S1). O_3_ of the TRY PC was adopted to represent a gradient including pre‐industrial (executed in C7), moderately increased (C6), and high (C5) concentrations. O_3_ of the PC TRY executed in C8 represented PC concentrations and was not changed. For preindustrial concentrations, a relative O_3_ concentration was calculated by normalizing O_3_ of TRY PC on the average annual O_3_ concentration and multiplying it by 10 ppb. For C6 and RCP2.6, hourly PC concentrations of O_3_ < 40 ppb were increased by +5 ppb, and O_3_ > 45 ppb were decreased by −5 ppb (as suggested by Hayes et al., 2019). For C5 and RCP8.5, O_3_ < 40 ppb was increased by +10 ppb and by +5 ppb for values between 40 and 50 ppb. O_3_ ≥ 50 ppb were reduced by −5 ppb in C5. TRY CO_2_ series were implemented in TUM*mesa* without adjustments, and air pressure was not regulated.

**TABLE 1:** Characteristics of the PC and FC (RCP2.6 and RCP8.5) TRY

		TRY	TRY experimental period	TUM*mesa* prescribed values
		Jan 01–Dec 31	Apr 16–Sep 29	
PC				
Air temperature, t_a_ (°C)	Range (mean)	−10.9 to 30.1 (8.2)	3.4 to 30.1 (14.5)	4.0 to 30.0 (14.6)
Relative humidity, rh (%)	Range (mean)	28.0 to 100.0 (77.9)	29.5 to 100.0 (70.6)	30 to 90.0 (67.7)
Air pressure, P (kPa)	Range (mean)	919 to 979 (956)	938 to 968 (956)	
CO_2_ (ppm)	Range (mean)	362 to 384 (375)	362 to 380 (370)	362 to 380 (370)
O_3_ (ppb)	Range (mean)	1 to 95 (29)	1 to 95 (39)	1 to 95 (39)
Global radiation, R_g_ (W m^−2^)	Mean (mean of daily max)	248 (447)	326 (650)	
PPFD (mol m^−2^) (µmol m^−2^ s^−1^)	Sum (% of TRY)	8048	5954	3934 (*66%)*
	Mean (mean of daily max)	508 (910)	671 (1340)	444 (672)
RCP 2.6				
Air temperature, t_a_ (°C)	Range (mean)	−6.3 to 28.9 (9.3)	−1.5 to 28.9 (15.3)	4.0 to 28.9 (15.5)
Relative humidity, rh (%)	Range (mean)	24.0 to 100.0 (78.8)	24.0 to 100.0 (72.3)	30.0 to 90.0 (68.3)
Air pressure, P (kPa)	Range (mean)	931 to 976 (958)	939 to 970 (956)	
CO_2_ (ppm)	Range (mean)	406 to 432 (421)	406 to 427 (416)	406 to 427 (416)
O_3_ (ppb)	Range (mean)	1 to 77 (22)	3 to 77 (30)	9 to 102 (42)
Global radiation, R_g_ (W m^−2^)	Mean (mean of daily max)	276 (483)	366 (723)	
PPFD (mol m^−2^) (µmol m^−2^ s^−1^)	Sum (% of TRY)	8946	6701	4150 (*62%*)
	Mean (mean of daily max)	564 (986)	755 (1492)	468 (694)
RCP 8.5				
Air temperature, t_a_ (°C)	Range (mean)	−5.9 to 32.5 (11.5)	0.7 to 32.5 (17.6)	4.0 to 30.0 (17.7)
Relative humidity, rh (%)	Range (mean)	28.0 to 100.0 (77.4)	30.4 to 100.0 (68.8)	30.0 to 90.0 (65.8)
Air pressure, P (kPa)	Range (mean)	928 to 979 (955)	929 to 969 (955)	
CO_2_ (ppm)	Range (mean)	903 to 959 (936)	903 to 949 (924)	903 to 949 (924)
O_3_ (ppb)	Range (mean)	1 to 96 (33)	1 to 96 (44)	11 to 95 (49)
Global radiation, R_g_ (W m^−2^)	Mean (mean of daily max)	266 (481)	351 (716)	
PPFD (mol m^−2^) (µmol m^−2^ s^−1^)	Sum (% of TRY)	8616	6426	4076 (64*%*)
	Mean (mean of daily max)	543 (981)	724 (1477)	460 (691)

Note

Range and mean (in brackets) of t_a_, rh, CO_2_, and O_3_ are shown for the simulated year, the experimental period and the derived set points for the TUM*mesa* climate chambers. The total sum of photosynthetic photon flux density (PPFD) over the respective period is shown. The highlighted values (in italics) in column 3 of PPFD sums represent the percentage of PPFD sums achieved in TUM*mesa* relative to the simulated data. For PPFD and R_g_, overall means and the means of daily maximum values (in brackets) are presented.

The prescribed hourly values were approached linearly in 1‐min‐steps in TUM*mesa*. The gradients were ~0.08°C min^−1^ (t_a_), 0.3% min^−1^ (rh), 0.3 ppb O_3_ min^−1^, and 0.7 ppm CO_2_ min^−1^ (only enrichment). The LEDs responded within seconds and no gradient was programed.

## 3 RESULTS

### 3.1 Test reference years

**FIGURE 5:**
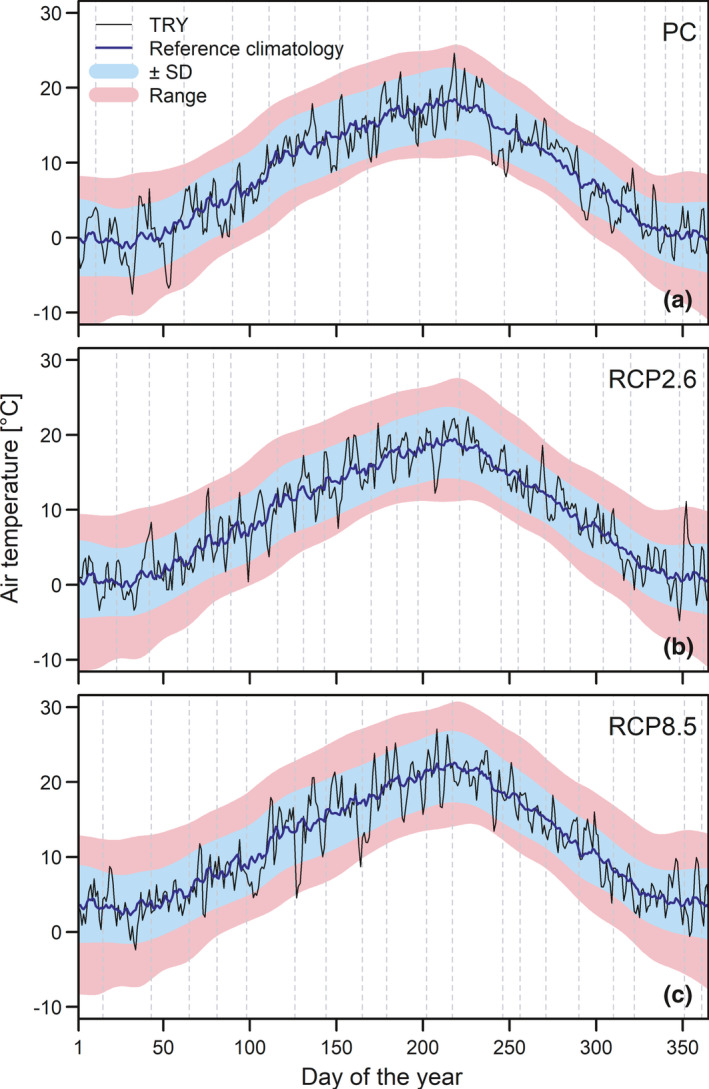
Annual course of air temperature of the TRYs generated for PC (a), RCP2.6 (b), and RCP8.5 (c) from reference climatologies by resampling of measured weather segments. RC for PC is the mean annual cycle recorded at the climate station "Spessart” (1987–2016). Reference climatologies for RCP2.6 and RCP8.5 are computed by adding the respective ensemble mean climate signal to RC. Smoothed ± SD of the mean annual cycle (PC) or of the ensemble means (RCP2.6, RCP8.5) is shaded blue for each RC. For PC, the range of 1987–2016 temperature records is shaded red (a). In (b) and (c), range is calculated by adding the respective ensemble mean climate signal to the range of RC. Data are presented in daily resolution. Dashed vertical lines indicate the transition between two consequent weather segments of the TRYs

**FIGURE 6:**
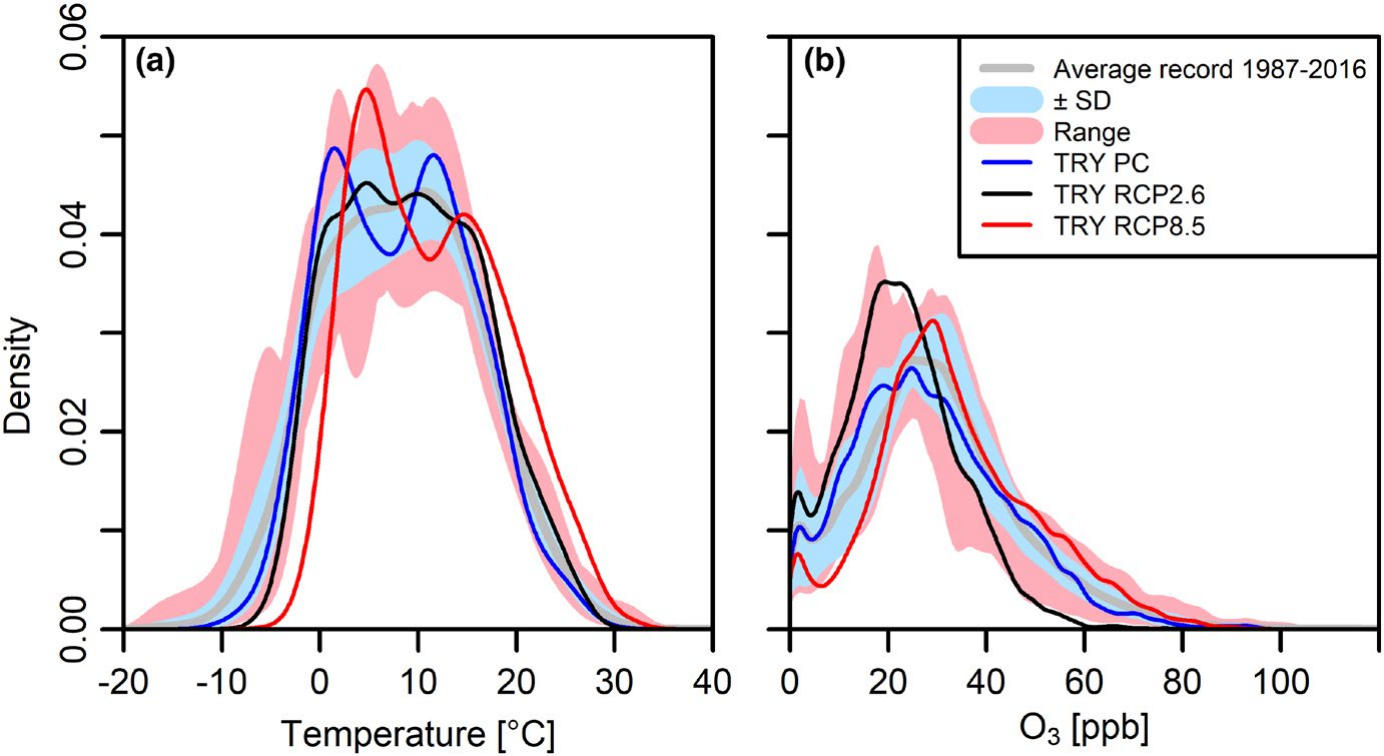
Probability density functions (PDFs) for temperature (a) and O_3_ (b) of the TRY. In addition, PDFs of the mean annual cycles of the reference period (1987–2016) recorded at “Spessart” ± *SD* are shown, as well as the historically observed range. PDFs were obtained by kernel density estimation using a bandwidth of 1.5

Three individual TRYs were generated for the climate station “Spessart” (summarized in Table 1). The TRYs consist of annual cycles for t_a_, rh, P, R_g_, O_3_, and CO_2_ in an hourly resolution, representing one possible year in each of the periods 1987–2016 (PC) and 2071–2100 (RCP2.6 and RCP8.5) (Figure 5). By recombining measured weather segments, the natural day‐to‐day variation of key meteorological variables is reintroduced (black line in Figure 5, only t_a_ is shown), which was lost in the reference climatologies (blue line) due to long‐term averaging of measured t_a_ for PC and the calculation of ensemble means for RCP 2.6 and RCP8.5. Average annual t_a_, rh, P, R_g_, and O_3_ of TRY PC are 8.2°C, 77.9%, 956 hPa, 248 W m^−2^, and 29 ppb and correspond well to the 1987–2016 long‐term averages of 8.3°C, 78.6%, 957 hPa, 255 W m^−2^, and 30 ppb measured at the climate station. When comparing TRY RCP2.6 to TRY PC, annual mean t_a_ increases by 1.1°C, CO_2_ increases by 12% from 375 ppm to 421 ppm and O_3_ declines by 24%. Increase of t_a_ in RCP8.5 is 3.3°C, CO_2_ increases 2.5‐fold to 936 ppm and O_3_ is elevated by 14%. The probability density function (PDF, Figure 6) for O_3_ of TRY RCP2.6 shows a pronounced increase in frequencies at around 20 ppb and highest values <80 ppb, whereas the PDF of O_3_ in TRY RCP8.5 indicates a considerable shift to higher values. Higher mean t_a_ in the RCPs are also reflected in the PDFs, which show a shift to higher minimum and maximum temperatures compared to TRY PC, but do not indicate higher t_a_ variability.

The percentage of time when daily minimum and maximum temperatures were below the 10th percentile of the reference period (TN10p, TX10p indices) decreased steadily from TRY PC to RCP2.6 and RCP8.5 (Table 2). The percentage of time when daily minimum and maximum temperatures were above the 90th percentile (TN90p, TX90p) increased minimally from TRY PC to TRY RCP2.6 (3.6%–6.8% in TN90p and 3.6%–6.3% in TX90p). However, the increase is most pronounced in TRY RCP8.5, indicating a two‐ to three‐fold increase in warm days and nights in the RCP 8.5 scenario at the end of the 21st century compared to the reference period of 1987–2016. The number of frost days decreased from 81 in TRY PC to 74 in TRY RCP2.6 and 21 in TRY 8.5, whereas the number of summer days increased from 17 to 25 and 54. Growing season length was 23 days and 71 days longer in RCP2.6 and RCP8.5 compared to TRY PC. The WSDI index indicated no warm spells for the TRYs, although WSDI was 5 ± 6 for the reference period at the climate station.

**TABLE 2:** Climate extreme indices of the TRYs for PC, RCP2.6, and RCP8.5 compared to the reference period 1987–2016 recorded at the climate station “Spessart”

Index			1987–2016	± SD	TRY PC	TRY RCP2.6	TRY RCP8.5
TN10p	Cool nights	Percentage of time when daily min temperature <10th percentile	10.6	± 3.9	3.8	1.6	0.5
TX10p	Cool days	Percentage of time when daily max temperature <10th percentile	10.7	± 4.9	5.2	1.4	0.3
TN90p	Warm nights	Percentage of time when daily min temperature >90th percentile	10.5	± 3.1	3.6	6.8	29.3
TX90p	Warm days	Percentage of time when daily max temperature >90th percentile	10.5	± 3.5	3.6	6.3	23.8
GSL	Growing season length	Annual count between first span of at least 6 days with TG > 5°C and first span after July 1 of 6 days with TG < 5°C	233	± 28	203	226	274
FD0	Frost days	Annual count when daily minimum temperature <0°C	85	± 18	81	74	21
SU25	Summer days	Annual count when daily max temperature >25°C	20	± 8	17	25	54
WSDI	Warm spell duration indicator	Annual count when at least six consecutive days of max temperature >90th percentile	5	± 6	0	0	0

### 3.2 Implementation of the TRY in TUM*mesa*

#### 3.2.1 Comparing prescribed to measured values

The TRYs were executed in TUM*mesa* on 161 of 167 operational days. Averaged over the entire experimental period, the maximum differences between measured and prescribed values were 0.1°C (t_a_), 0.8% (rh), 13 ppm CO_2_ (at PPFD > 100 µmol m^−2^ s^−1^), 4 ppb O_3_, and 113 µmol m^−2^ s^−1^ PPFD (Table 3). Prescribed values were approached in one‐min‐steps, resulting in 1,854,720 data pairs for each parameter. Data losses were below 1%.

**TABLE 3:** Mean values of climate parameters prescribed in TUM*mesa* compared to the mean values measured in each chamber (C1–C8)

	Prescribed values	Measured values							
PC		C5		C6		C7		C8	
Air temperature, t_a_ (°C)	14.6	14.6	*0.0*	14.5	−*0.7*	14.6	*0.0*	14.6	*0.0*
Relative humidity, rh (%)	67.7	68.4	+*1.0*	68.4	+*1.0*	68.1	+*0.6*	68.5	+*1.2*
CO_2_ (ppm)	370	376	+*1.6*	374	+*1.1*	382	+*3.2*	381	+*3.0*
O_3_ (ppb)	39							41	+*5.1*
PPFD (µmol m^−2^ s^−1^)	444	512	+*16.4*	512	+*15.3*	512	+*15.3*	543	+*22.3*

RCP2.6		C3		C4					
Air temperature, t_a_ (°C)	15.5	15.5	+*0*.*0*	15.4	−*0.6*				
Relative humidity, rh (%)	68.3	68.9	+*0.9*	68.7	+*0.6*				
CO_2_ (ppm)	416	411	−*1.2*	413	−*0.7*				
O_3_ (ppb)	42	45	+*7.1*	44	+*4.8*				
PPFD (µmol m^−2^ s^−1^)	468	570	+*21.8*	551	+*17.7*				

RCP8.5		C1		C2					
Air temperature, t_a_ (°C)	17.7	17.8	+*0.6*	17.8	+*0.6*				
Relative humidity, rh (%)	65.8	66.0	+*0.3*	65.9	+*0.2*				
CO_2_ (ppm)	924	918	−*0.6*	911	−*1.4*				
O_3_ (ppb)	49	53	+*8.2*	52	+*6.1*				
PPFD (µmol m^−2^ s^−1^)	460	559	+*21.5*	573	+*24.6*				

Note

C1 and C2 executed TRY RCP8.5, C3 and C4 TRY RCP2.6. TRY PC was executed in C5–C8, but the corresponding O_3_ series was implemented only in C8. Numbers in italics indicate the relative deviation from prescribed values (in percent). Mean PPFD was calculated for daytime values and CO_2_ values were excluded where PPFD was below 100 µmol m^−2^ s^−1^.

**FIGURE 7:**
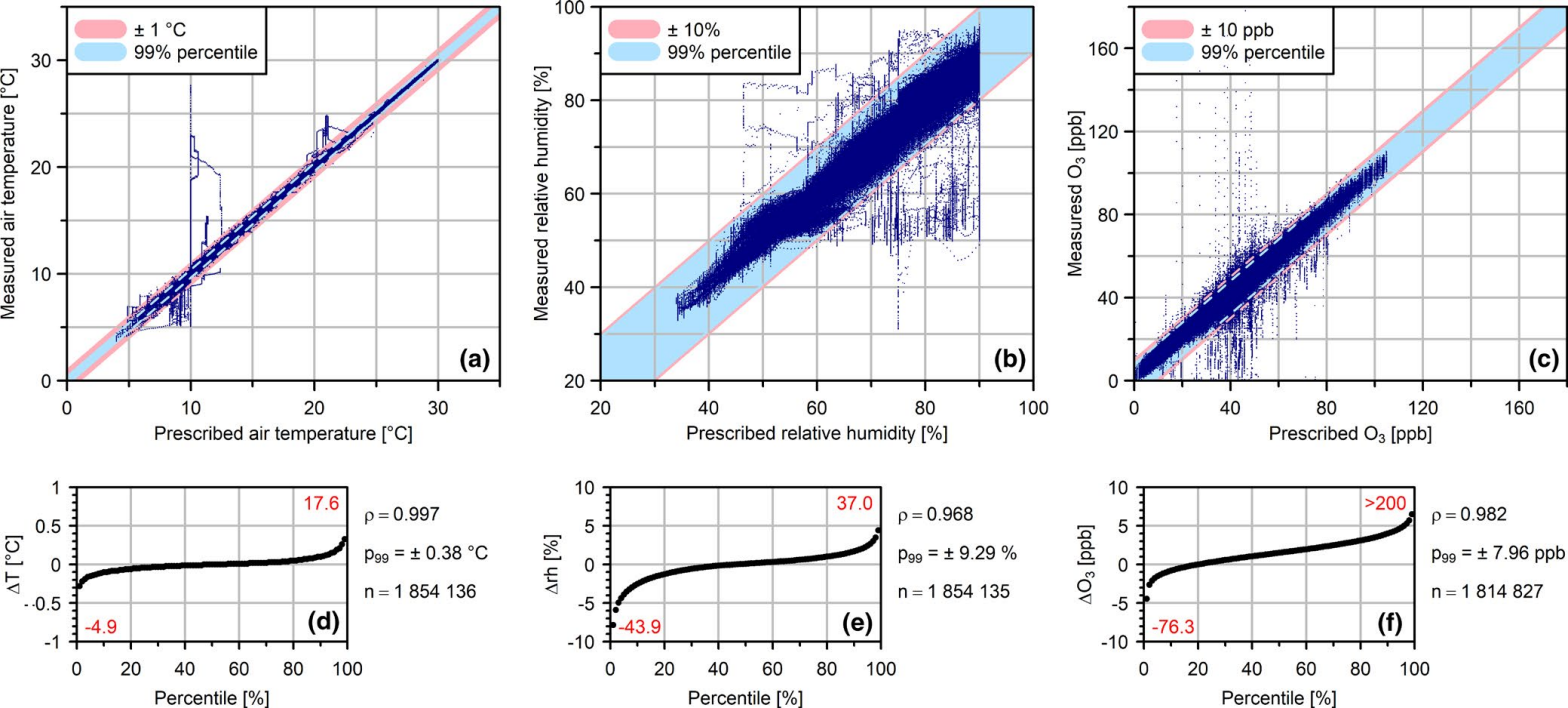
Deviation of measured air temperature (a), relative humidity (b), and ozone concentration (c) from prescribed values. Underlying values have a resolution of 1 min. The tolerated deviation is shaded red; the 99% percentile of the absolute difference (listed as p_99_) between prescribed and measured values is shaded blue. Pearson's correlation coefficient (ρ) and sample size (n) are listed. Percentiles of difference (Δ) between prescribed and measured values within the tolerated deviation are shown in (d), (e), and (f). Red numbers indicate 0% (bottom left) and 100% (top right) percentiles

For t_a_, 99% of measured values were within a ± 0.38°C deviation of prescribed values (Figure 7a). Deviations exceeding +1°C (<0.2% of all records) were caused mainly by failure of the adiabatic precooling system or by a defective precooling of the outside air inlet. Deviations of more than −1°C (<0.2% of all records) were related to a malfunction of the chamber heating register.

For rh, 99% of measured values deviated less than ± 9.3% from prescribed values (Figure 7b). Most of the deviations greater than tolerated (± 10%) were caused by an insufficient supply of feed water to the steam generators or by insufficient heating voltages.

During the 161 days of operation, 99% of measured O_3_ values deviated < ± 8.0 ppb from prescribed values (Figure 7c). However, O_3_ fumigation in each chamber had to be deactivated for 83 h (i.e., 2.1% of operation time) due to a failure of the ozone analysis, which led to an unregulated ozone fumigation in two chambers (C1 and C3) over a period of 8 hr. Peak concentrations during this period exceeded 200 ppb. The ozone fumigation was switched off until the malfunction was permanently corrected. Opening the chamber doors and working in the chambers generally resulted in a reduction of O_3_ concentration, which exceeded the tolerated deviation of ± 10 ppb in 0.5% of records; 81% of all records showed positive deviation from the target values (0.2% of records exceeding the tolerated range), due to a frequent control‐related overshoot during up‐regulation of O_3_ concentrations.

The difference in measured PPFD to prescribed values (Table 3) is explained by that fact that LEDs are controlled in relative steps from 0% to 100%. The relative intensities are related to a PPFD in 100 cm distance from the light source. Light intensity increases with decreasing distance to the source. In this study, the PAR sensor was installed in 70 cm distance from the LEDs to avoid shading by the trees.

#### 3.2.2 Control of CO_2_ concentration

**FIGURE 8:**
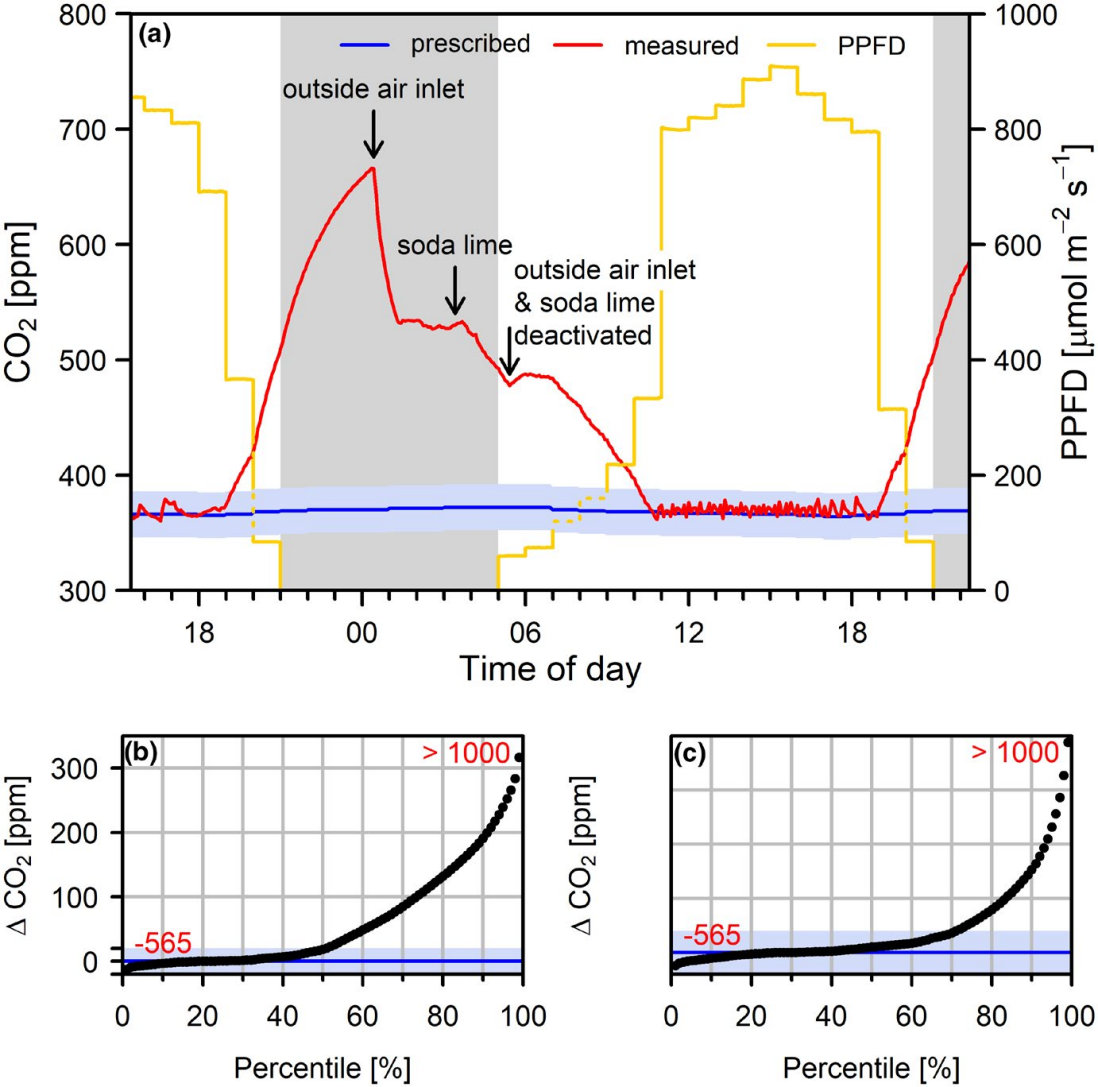
Typical diurnal course of CO_2_ concentrations (a) in the PC scenario. The tolerated deviation from prescribed values is shaded blue. Arrows indicate (i) start of outside air inlet, (ii) activation of CO_2_ removal via soda lime and (iii) the deactivation of both outside air inlet and CO_2_ removal. Percentiles of difference (Δ) between actual values and set points are shown in (b). In (c), only data pairs are considered for which the door was closed for at least one full hour and PPFD was above 10 µmol m^−2^ s^−1^. The tolerated deviation from prescribed values is shaded blue. Red numbers in (b) and (c) indicate 0 (bottom left) and 100% (top right) quantiles

In total, 49.8% of all CO_2_ records deviated more than the tolerated ± 20 ppm from prescribed values (Figure 8b). Only 0.5% of all records were below −20 ppm of the target, whereas 49.3% of the deviations exceeded +20 ppm; 2.0% of all positive deviations could be attributed to people entering and working in the chambers. The main reason for the larger number of positive deviations was nocturnal plant respiration and an insufficient CO_2_ removal by soda lime. During the nonphotoperiod, 99.7% of records were above the tolerated range as plant respiration caused massive increase of CO_2_ levels (Figure 8a), frequently exceeding 700 ppm. To ensure that CO_2_ concentrations during the day were not excessively influenced by increased nocturnal values outside air with lower CO_2_ concentration was blown into the chamber for 3 h before daybreak in addition to active CO_2_ removal by soda lime (Figure 8a). This enabled us to maintain CO_2_ within the tolerated range for 71.2% of values when there was no influence of human respiration and PPFD was above 10 µmol m^−2^ s^−1^ (Figure 8c).

#### 3.2.3 Homogeneity among chambers

**FIGURE 9:**
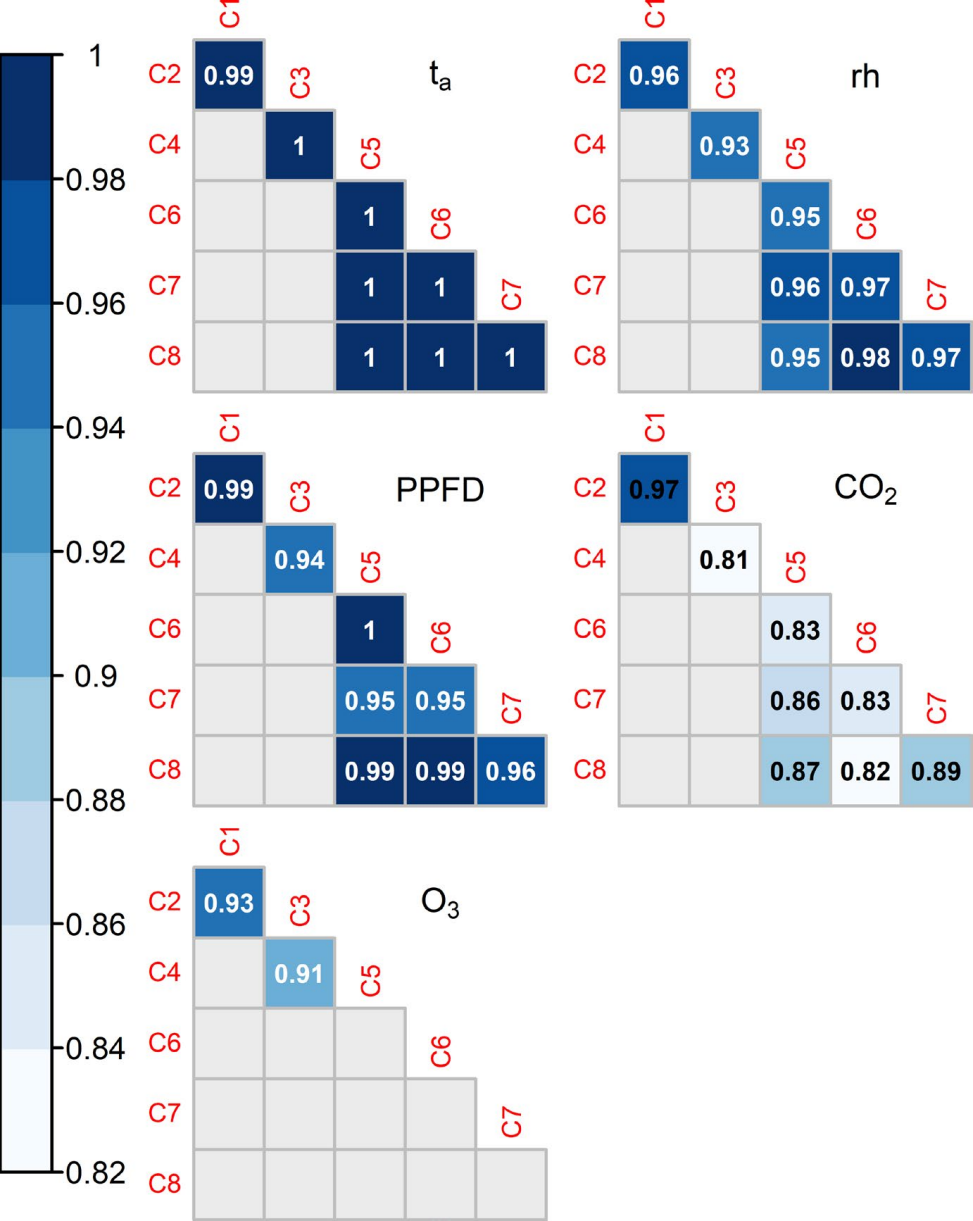
Pearson's correlation coefficients between measured climate variables among climate chambers (C1–C8) executing identical time series. Identical time series of air temperature (t_a_), relative humidity (rh), light (PPFD), and CO_2_ were executed in chambers C1/C2, C3/C4, and C5/C6/C7/C8. Identical time series of O_3_ were executed in chambers C1/C2 and C3/C4. Correlation matrices are based on 214,505–231,785 data points per chamber

To verify the homogeneity among chambers executing identical time series, correlation matrices were calculated for the parameters t_a_, rh, O_3_, CO_2_, and PPFD using Pearson's correlation coefficient (r, Figure 9). The correlation coefficients for t_a_ were 1, except between C1/C2, where it was 0.99. Over 99% of t_a_ data pairs show less than a ± 1°C deviation among chambers. Minimum r was 0.93 for rh and 0.91 for O_3_, where 98% and 99% of all data pairs were within the tolerated deviation of ± 10% and ± 20 ppb, respectively. Minimum r was 0.94 for PPFD and more than 90% of all data pairs showed less than ± 50 µmol m^−2^ s^−1^ PPFD deviation among the chambers. However, PAR sensors were sometimes shaded by the growing canopy, such that the actual deviations among individual chambers can be assumed to be lower. For CO_2_, lowest r was 0.88. Nevertheless, 80% of all relevant CO_2_ data pairs deviated less than ± 20 ppm among chambers that performed identical time series. With PPFD > 10 µmol m^−2^ s^−1^ and doors closed, even 89% of deviations among chambers were within ± 20 ppm.

## 4 DISCUSSION

The methodological approach presented here enables the generation of regionalized climate series with high temporal resolution for implementation in controlled environment experiments. Starting from downscaled climate simulations of the EURO‐CORDEX and ReKliEs‐DE ensembles, possible annual courses for PC and FC scenarios were generated by resampling from historical records. The obvious advantage of this method is that consistent segments of all meteorological variables available for a location are combined in such a way that the statistical properties of a simulated climate scenario are adequately reproduced. By incorporating weather data of simultaneously measured t_a_, rh, R_g_, and O_3_, the natural intra‐annual variability and covariance between these variables are preserved. Annual averages of t_a_, rh, P, R_g,_ and O_3_ of the PC TRY correspond well to the 30‐year averages from the reference period recorded at the climate station. The t_a_ increases of 1.1 and 3.3°C in the RCP2.6 and RCP8.5 TRYs are within the range predicted by the CMIP5 model simulations for the end of the 21st relative to 1986–2005 (i.e., 0.3–1.7°C for RCP2.6 and 2.6–4.8°C for RCP8.5, IPCC, 2013).

O_3_ series for the FC TRYs were adjusted relative to the ensemble mean ground‐level O_3_ concentrations simulated by Sicard et al. (2017) for the northern hemisphere in 2100. The authors simulated their data using the model ensemble of the Atmospheric Chemistry and Climate Model Intercomparison Project (ACCMIP, Lamarque et al., 2013). Naturally, however, the diurnal and seasonal dynamics of regional O_3_ are not well represented on such a rough scale. They are rather driven by small‐scale topography, land use and settlement density, as well as by intercontinental long‐distance transport. The future development of these dynamics is difficult to predict. Mitigation measures to reduce CH_4_ and NO_X_ were shown to have the largest contribution to changes in surface O_3_ (Sicard et al., 2017), but uncertainties arise concerning their future regional and local behavior due to their long lifespan in the hemispherical background (Turnock et al., 2019). Models are further complicated by interactions between VOCs and O_3_ formation (Calfapietra et al., 2013; Peñuelas & Staudt, 2010), intercontinental O_3_ transport (Derwent et al., 2004; Volz‐Thomas et al., 2003) and influx of stratospheric ozone (Kawase et al., 2011). Thus, regional development of tropospheric O_3_ concentrations particularly depends on a number of interacting factors, the prediction of which is subject to many uncertainties.

The atmospheric CO_2_ concentration was not measured at the “Spessart” station. Assuming that the CO_2_ concentration at the station is influenced on a rather large scale, data from the nearest CO_2_ monitoring site “Schauinsland” were used. Unlike a forest canopy, atmospheric CO_2_ at the station is largely unaffected by convective boundary layer effects and canopy dynamics. Therefore, pronounced diurnal CO_2_ variation—as observed in forest canopies (Murayama et al., 2003)—are not represented in the TRYs. However, due to insufficient CO_2_‐removal from the chamber atmosphere, nocturnal CO_2_ increase due to respiration processes was also established in the climate chambers, although unregulated and on a higher level than expected for natural forest ecosystems. By temporarily supplying outside air in combination with the soda lime columns, the targeted CO_2_ concentrations could be achieved for more than 70% of the photoperiod.

Precipitation was not considered in the generation of TRYs because the objectives of the valORTree project required adequate soil moisture availability. Nevertheless, to account for a possible reduction in future precipitation, FC TRYs were replicated with periods of reduced soil moisture in the valORTree project. We did, however, not derive precipitation from the ReKliEs‐De ensemble, because the simulations of summer precipitation performed by Hübener et al. (2017) indicate huge uncertainties, ranging −30% to +5% for RCP2.6 and −60% to +20% for RCP8.5 for the end of the 21st century relative to 1971–2000.

The TRYs were generated to represent one possible annual cycle for average 30‐years‐periods of present and FC. It is well agreed upon that FC will not only be characterized by changes in mean meteorological quantities, but ecosystems will be subjected to changes in the magnitude or frequency of extreme events (Harris et al., 2018; Jentsch et al., 2007; Rahmstorf & Coumou, 2012). By calculating multimodel means, variability and extreme events are systematically smoothed out. Intra‐annual variation was reintroduced to our TRYs by resampling from the historical record, and interannual variability can be introduced by starting the recombination process for a proceeding TRY from a different weather segment. However, as highlighted by Boé et al. (2006), resampling is incapable of providing values outside the range of those already observed. Nevertheless, resampling enabled us to produce TRYs for the RCP2.6 and RCP8.5 scenarios that are substantially characterized by decreases in the low temperature related indices TN10p, TX10p, and FD0 as well as increases in the high temperature related indices TN90p, TX90p, and SU25 that are expected to be observed in future. As expected, growing season lengths increased in the FC TRYs. However, the WSDI index was 0 for all TRYs, indicating that extreme heat periods did not occur in the TRYs. The t_a_ distribution of the TRYs (Figure 6) indicates a clear shift to higher temperatures in the RCPs, but no increase in the variability is present as would be evident by a broader distribution.

Recently, Vanderkelen et al. (2020) introduced a method for forcing the UHasselt Ecotron units directly with 3‐hourly output of a single best‐performing GCM:RCM combination from the EURO‐CORDEX ensemble. By identifying a single model projection, the method respects not only the covariation between climatic variables but also their projection in variability, as well as possible extreme events. However, single models are strongly influenced by uncertainty in climate predictions resulting from structural differences in the GCMs as well as uncertainty due to variations in initial conditions or model parameterization (Semenov & Stratonovitch, 2010). These uncertainties could be overcome by applying several single‐model projection in parallel in an ecological experiment (as suggested by Thompson et al., 2013) or by running climate simulations as well as experiments for multiple years and thereby catching the inter‐annual variation of climate variables (as performed by Vanderkelen et al., 2020). However, most CEF experimental designs are limited in duration as well as the number of available experimental units. Considering this, the methodological approach presented here is a suitable alternative that respects changes in mean quantities of FC scenarios as well as co‐variability between multiple climate drivers and intra‐annual variability. Additionally, periods of extreme weather conditions can be represented in the TRYs following Roy et al. (2016) by superimposing, for example, a period of high temperature on the TRYs.

## 5 CONCLUSION

The TRYs generated with the methodology described here capture possible changes in the mean values of important meteorological drivers while maintaining intra‐annual variability and covariability between the multiple drivers. By calculating multimodel means, the method is, however, not capable of reproducing extreme events in a sophisticated way, and changes in climate extreme indices for our RCP TRYs represent the shift in mean values (as indicated in the PDFs) rather than the presence of extreme events. Nevertheless, the method produces dynamic multivariate climate series for the implementation in ecological CEF studies that focus on general impacts of climate change on ecological systems on a regional scale. The TRYs are a suitable alternative to CEF climate simulations based on simple day/night cycles and incremental manipulations of single climate variables. The TRYs were adequately simulated in the TUM*mesa* CEFs, with particularly good reproduction of absolute values and high‐resolution dynamics of temperature, relative humidity, ozone, and light.

## CONFLICT OF INTEREST

The authors declare that the research was conducted in the absence of any commercial or financial relationships that could be construed as a potential conflict of interest.

## AUTHOR CONTRIBUTIONS

**Bálint Jákli:** Investigation (lead); Project administration (supporting); Visualization (lead); Writing‐original draft (lead); Writing‐review & editing (equal). **Roman Meier:** Data curation (equal); Investigation (supporting); Validation (equal); Writing‐review & editing (equal). **Ulrike Gelhardt:** Methodology (equal); Writing‐original draft (supporting); Writing‐review & editing (equal). **Margaret Bliss:** Writing‐original draft (supporting); Writing‐review & editing (equal). **Ludger Grünhage:** Conceptualization (equal); Methodology (equal). **Manuela Baumgarten:** Conceptualization (equal); Funding acquisition (lead); Project administration (lead); Writing‐original draft (supporting); Writing‐review & editing (equal).

## Supporting information

Appendix S1

## Data Availability

The TRY for PC, RCP2.6 and RCP8.5 are available on Dryad (DOI https://doi.org/10.5061/dryad.h18931zn5).
