# Supplementary material for: Regionalized dynamic climate series for ecological climate impact research in modern controlled environment facilities
Source: Ecol Evol. 2021 Nov 20;11(23):17364–80. doi: 10.1002/ece3.8371 (PMC8668799; doi:10.1002/ece3.8371)
Supplement: Supplementary file 1 — Appendix S1 [file ECE3-11-17364-s001.docx]

APPENDIX

Table A1: Requirements for in-use performance of TUM*mesa* climate chambers within the distances of 150 mm from the walls, 200 mm from the ceiling and 650 mm above the floor. Accuracy is valid for both temporal and spatial deviation of process parameters in steady state operation.

|  | temperature control | range | 4 – 30 °C without lighting  10 – 30 °C with lighting  up to 40 °C without accurate humidity control |
| --- | --- | --- | --- |
|  |  | accuracy | ± 1 K in steady state |
|  | relative humidity control | range | 30 – 90 % without lighting  30 – 75 % with lighting |
|  |  | accuracy | ± 10 % in steady state |
|  | dew point temperature | range | 5 – 28 °C |
|  | supply air |  | circulating air or 30 – 100 m^3^ h^-1^ |
|  | exhaust air |  | circulating air or 30 – 100 m^3^ h^-1^ |
|  | wind speed inside chamber |  | < 0.1 m s^-1^ |
|  | CO_2_ control | range | ambient – 3000 ppm |
|  |  | accuracy | ± 20 ppm |
|  | CO_2_ analyzer (URAS26, ABB Automation GmbH, Germany) | range | 0 – 5000 ppm |
|  |  | accuracy | ± 2 % |
|  | CO_2_ fumigation |  | 0 – 20 l min^-1^ |
|  | addition of CO_2_-free air |  | 30 l min^-1^ with 10 – 30 ppm residual concentration |
|  | ^13^CO_2_ fumigation |  | 1 – 20 ml min^-1^ |
|  | O_3_ control | range | 0 to 200 ppb |
|  |  | accuracy | ± 10 ppb |
|  | O_3_ analyzer (BMT 932, BMT  Messtechnik GmbH, Germany) | range | 0 to 200 ppb |
|  |  | accuracy | ± 10 ppb |
|  | O_3_ generation |  | 4 g h^-1^ for each climate chamber |
|  | LED spectrum | range | 340 – 730 nm (10 individually controlled LED bands) |
|  |  | band maxima | 340, 365 - 405 , 430, 490, 530, 630, 660, 730 nm  cool white band (4000 K), warm white band (6500 K) |
|  | photosynthetic photon flux density (PPFD) | intensity | > 1500 µmol m^-2^ s^-1^ in 1 m distance from panel  default operation mode at 55 % capacity (max > 800 µmol m^-2^ s^-1^) |
|  |  | homogeneity | ± 10 % horizontal  ± 20 % vertical |


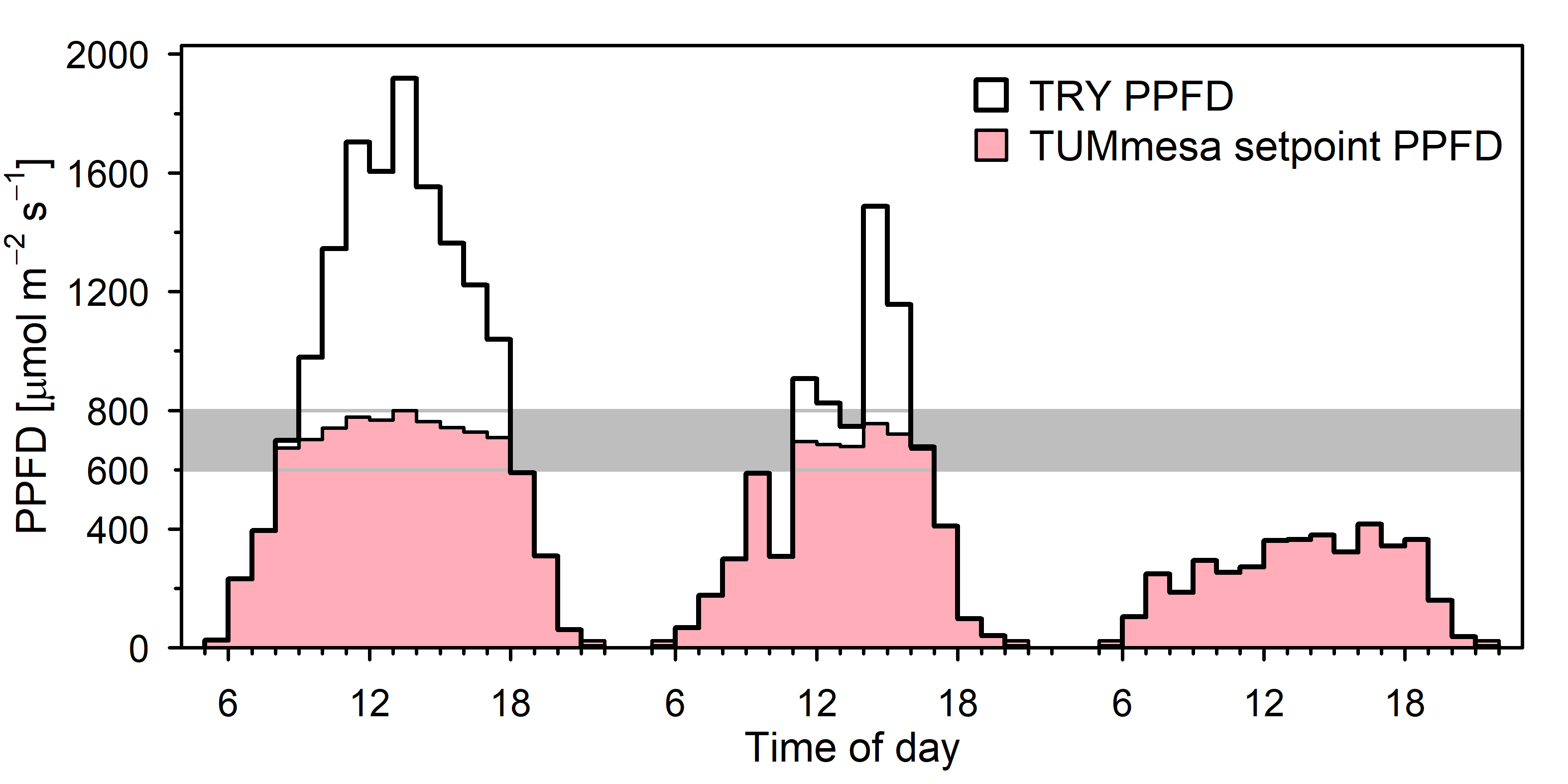


Fig. A1: Adaptation of light intensities to the TUMmesa requirements. PPFD of the Test Reference Years (TRY) between 600 µmol m^-2^ s^-1^ and the present climate (PC) maximum intensity of 2030 µmol m^-2^ s^-1^ were scaled to the range from 600 to 800 µmol m^-2^ s^-1^ (shaded grey). Below 600 µmol m^-2^ s^-1^ the prescribed values follow the simulated course, except for PPFDs < 24 µmol m^-2^ s^-1^ which were increased to 24 µmol m^-2^ s^-1^ due to minimum requirements of the LEDs. Three different diurnal courses (DOY 191, 195 and 196) of the PC TRY are shown.

Fig. A2: Possible combinations of a Global Climate Model (GCM) with a Regional Climate Model (RCM) or an Empirical Statistical Regionalization Method (ESD) for RCP2.6 and RCP8.5. Simulations within EURO-CORDEX and ReKliEs DE are shaded blue and red, respectively. Model combinations accepted for the present project are highlighted. Adopted from Hübener et al. (2017).


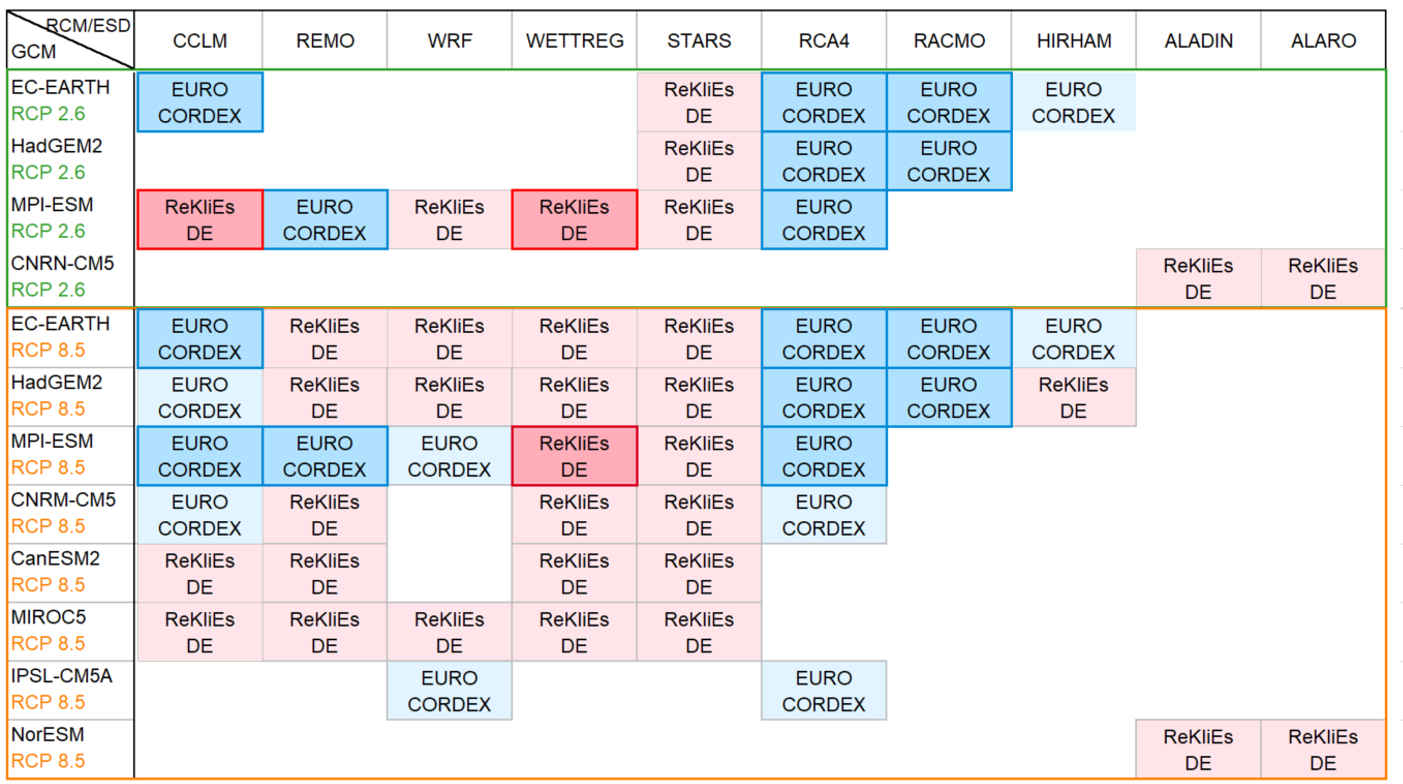


Combinations that were technically excluded:

- Climate projections using IPSL-CM5A: by analyzing historical climate simulations and observational data, this GCM has been classified as an outlier (McSweeney et al., 2015)
- Climate projections using WRF and HIRHAM: no variation of atmospheric greenhouse gas concentrations included (Jerez et al., 2018)
- Climate projections with STARS: not recommended by the Potsdam Institute for Climate Research (PIK) for use with RCP8.5 for projections later than 2060
- Climate projections with ALADIN and ALARO: These RCMs are not provided for the shared 11° grid of EURO-CORDEX and ReKliEs-De

Tab. A2.1: Abbreviations for Global Climate Models (GCM)

| Abbreviation | Model name | Institute |
| --- | --- | --- |
| EC-EARTH | ICHEC-EC-EARTH | EC-EARTH Consortium |
| HadGEM2 | MOHC-HAdGEM2-ES | Met Office Hadley Centre |
| MPI-ESM | MPI-M-MPI-ESM-LR | Max Planck Institute for Meteorology |
| CNRM-CM5 | CNRM-CERFACS-CNRM-CM5 | Centre National de Recherches Meteorologiques / Centre Europeen de Recherche et Formation Avancees en Calcul Scientiﬁque |
| CanESM2 | CCCMa-CanESM2 | Canadian Centre for Climate Modelling and Analysis |
| MIROC5 | MIROC-MIROC5 | Atmosphere and Ocean Research Institute(University of Tokyo) / National Institute for Environmental Studies / Japan Agency for Marine-Earth Science and Technology |
| IPSL-CMA5A | IPSL-IPSL-CMA5A-MR | Institute Pierre-Simon Laplace |
| NorESM | NorESM1-M | Bjerknes Centre for Climate Research / Norwegian Meterological Institute / Dept. of Geosciences (University of Oslo) / Geophysical Institute (University of Bergen) |

Tab. A2.2: Abbreviations for Regional Climate Model (RCM) and Empirical Statistical Regionalization Method (ESD)

| Abbreviation | Model name |
| --- | --- |
| CCLM | CCLM 4-8-17 |
| REMO | REMO |
| WRF | IPSL-INERIS-WRF |
| WETTREG | WETTREG2013 |
| STARS | STARS3 |
| RCA4 | SMHI-RCA4 |
| RACMO | KNMI-RACMO22E |
| HIRHAM | DMI-HIRHAM5 |
| ALADIN | ALADIN |
| ALARO | ALARO-0 |

References

Jerez, S., López-Romero, J. M., Turco, M., Jiménez-Guerrero, P., Vautard, R., & Montávez, J. P., 2018. Impact of evolving greenhouse gas forcing on the warming signal in regional climate model experiments. Nature Communications, 9(1), 1–7. https://doi.org/10.1038/s41467-018-03527-y

Hübener, H., Bülow, K., Fooken, C., Früh, B., Hoffmann, P., Höpp, S., Keuler, K., Menz, C., Mohr, V., Radtke, K., et al., 2017. ReKliEs-De Results Report [ReKliEs-De Ergebnisbericht, in German]; World Data Center for Climate (WDCC) at DKRZ: Hamburg, Germany.

McSweeney, C. F., Jones, R. G., Lee, R. W., & Rowell, D. P. , 2015. Selecting CMIP5 GCMs for downscaling over multiple regions. Climate Dynamics, 44(11–12), 3237–3260. https://doi.org/10.1007/s00382-014-2418-8
